# Intermittent Fasting Regimes Reduce Gingival Inflammation: A Three‐Arm Clinical Trial

**DOI:** 10.1111/jcpe.14151

**Published:** 2025-03-09

**Authors:** C. L. Pappe, J. Maetschker, S. Dujardin, B. Peters, O. Pivovarova‐Ramich, F. Kandil, A. Michalsen, C. Breinlinger, N. Steckhan, D. Koppold, H. Dommisch

**Affiliations:** ^1^ Department of Periodontology, Oral Medicine and Oral Surgery Charité – Universitätsmedizin Berlin, Corporate Member of Freie Universität Berlin, and Humboldt‐Universität zu Berlin Berlin Germany; ^2^ Department of Prosthetic Dentistry, University School of Dental Medicine Martin Luther University Halle‐Wittenberg Halle Germany; ^3^ Department of Molecular Metabolism and Precision Nutrition German Institute of Human Nutrition Potsdam‐Rehbruecke Nuthetal Germany; ^4^ German Center for Diabetes Research (DZD) München‐Neuherberg Germany; ^5^ Department of Endocrinology and Metabolism Charité – Universitätsmedizin Berlin, Corporate Member of Freie Universität Berlin, and Humboldt‐Universität zu Berlin Berlin Germany; ^6^ Institute of Social Medicine, Epidemiology and Health Economics, Charité–Universitätsmedizin Berlin Corporate Member of Freie Universität Berlin and Humbolt‐Universität zu Berlin Berlin Germany; ^7^ Department of Internal and Integrative Medicine Immanuel Hospital Berlin Berlin Germany; ^8^ Digital Health‐Connected Healthcare, Hasso Plattner Institute University of Potsdam Potsdam Germany; ^9^ Evidence‐Based Digital Diabetology, Medical Faculty Carl Gustav Carus, Department of Medicine III, Prevention and Care of Type 2 Diabetes Technical University of Dresden Dresden Germany; ^10^ Charité – Universitätsmedizin Berlin, Corporate Member of Freie Universität Berlin, Charité Competence Center for Traditional and Integrative Medicine (CCCTIM) Humboldt‐Universität zu Berlin and Berlin Institute of Health Berlin Germany; ^11^ Department for Prevention and Care of Diabetes, Department of Medicine III, Faculty of Medicine Carl Gustav Carus Technische Universität Dresden Dresden Germany

**Keywords:** Bahá'í fasting, bleeding on probing, crevicular fluid, experimental gingivitis model, gingival inflammation, intermittent fasting/16:8 fasting

## Abstract

**Aim:**

To evaluate the effect of religious Bahá'í dry fasting (BF) or 16:8 time‐restricted eating (TRE) compared with a regular diet (CG) on periodontal parameters during a modified experimentally induced gingivitis.

**Material and Methods:**

All participants were asked to refrain from oral hygiene (3 sextant) for 9 days (T1–T2) and were followed for a total of 19 days (T3) while adhering to fasting or a regular diet and resuming oral hygiene. The primary outcome was bleeding on probing in the test sextant (BOP_s), Rustogi plaque index (RPI), gingival crevicular fluid (GCF), blood pressure (BP), body weight (BW), HbA1c and C‐reactive protein (CRP) were measured (T1–T3) and ANCOVA and post hoc comparison were applied.

**Results:**

Sixty‐six healthy participants were recruited. Forty‐three were randomly assigned to TRE (*n* = 22) and CG (*n* = 21), while 23 followed BF, avoiding food and drinks during the day. At T2, BF demonstrated significantly less increase in BOP_s, and GCF increased in CG only. Analysis revealed significant differences in change for BOP_s between BF and CG (−9.48% [−17.18; −1.79]) and BF and TRE (−9.19% [−15.07; −3.32]) as well as for GCF between BF and CG (−0.06 μL [−7.22; −0.66]) and TRE and CG (−0.08 μL [−0.17; −0.00]).

**Conclusion:**

This study indicates beneficial effects of different fasting protocols on oral experimental gingivitis and metabolic parameters, but results are limited by randomisation issues and potential bias in the BF group.

## 1 Introduction

Oral inflammatory diseases, such as gingivitis and periodontitis, rank among the most prevalent inflammatory conditions globally (Billings et al. 2018; Kassebaum et al. 2017). Their aetiology is multifaceted, with behavioural factors like unhealthy diets playing an important role in pathogenesis (Chapple et al. 2017; Martinon et al. 2021; Meyle and Chapple 2015). Conversely, ‘healthy’ dietary interventions have been shown to reduce periodontal inflammatory parameters in experimental gingivitis settings (Bartha et al. 2022; Baumgartner et al. 2009; Woelber et al. 2019); however, evidence for periodontal treatment remains insufficient referring to recent guidelines (Ramseier et al. 2020; Sanz et al. 2020). A popular dietary intervention is intermittent fasting (IF) due to its metabolic‐regulating effects, beyond merely depleting unhealthy foods (Varady et al. 2022). Reductions in body weight, markers of oxidative stress and fat mass are well documented, whereas evidence for effects on blood pressure, lipids, HbA1c and inflammatory markers is inconsistent (Varady et al. 2022). IF involves alternating periods of caloric restriction (CR) or total fasting with fasting windows ranging up to 48 h, followed by *ad libitum* eating. Time‐restricted eating (TRE), a subtype of IF, requires fasting for a minimum of 14 h per day (Koppold et al. 2024). While the optimal fasting duration and timing of TRE remain inconclusive (Petersen et al. 2022), the main fasting period typically occurs overnight. In contrast, religious fasting practices, such as Ramadan and Bahá'í fasting, represent intermittent daytime dry fasting, with fasting during daylight hours and eating at night (Koppold‐Liebscher et al. 2021).

Research on religious IF has shown short‐term favourable effects on lipid profiles, weight and blood pressure (Koppold‐Liebscher et al. 2021; Kul et al. 2014). However, studies examining the impact of religious IF on oral health are scarce. People performing Ramadan fasting tend to visit dental practices less frequently due to concerns about breaking the fast (Shaeesta et al. 2015). Moreover, self‐performed oral hygiene may be compromised during the day, potentially leading to increased gingival bleeding and plaque index (Narayanan et al. 2016). Studies suggest that religious IF affects saliva composition, including an increased pH; however, evidenced data on periodontal and microbial changes are currently lacking (Aripin et al. 2024).

Studies investigating the effects of fasting/CR outside religious contexts on periodontal health are scarce, and when performed in humans, contradictory results were reported. An increased gingival index and decreased plaque levels were observed in obese men participating in a water‐only fasting regimen with vitamin supplements for 44 days (Squire and Costley 1976). Whereas a four‐week weight control programme, involving CR and exercise, showed no changes in bleeding on probing (BOP) but a reduction in pro‐inflammatory biomarkers in gingival crevicular fluid (GCF) (Park et al. 2015). Further, IF combined with a Mediterranean diet over 6 months could demonstrate a significant reduction in BOP and pocket depth in 14 periodontal patients without subgingival instrumentation (Lira‐Junior et al. 2024). Our group demonstrated an anti‐inflammatory effect of prolonged fasting on periodontal parameters in a female cohort with metabolic syndrome and periodontitis, independent of plaque levels (Pappe et al. 2021).

Compared with prolonged fasting, TRE represents an easy‐to‐implement regime, while BF, performed annually in March, provides a suitable cohort for studying dry fasting practices. Experimentally induced gingivitis in healthy patients minimises lifestyle, disease and medication influences, allowing a more focused investigation of the intervention. Therefore, the objective of this study was to investigate the effects of BF (avoiding food and drinks during the day) and TRE (16 h fasting, 8 h eating window) on periodontal parameters during locally induced experimental gingivitis (test sextant 3) when compared with a regular diet. We hypothesised that both fasting regimens would lead to reduced BOP_s levels as a primary outcome (differences between groups at T2, with T1 as covariates) compared with controls.

## 2 Material and Methods

### 2.1 Ethics and Study Design

**FIGURE 1:**
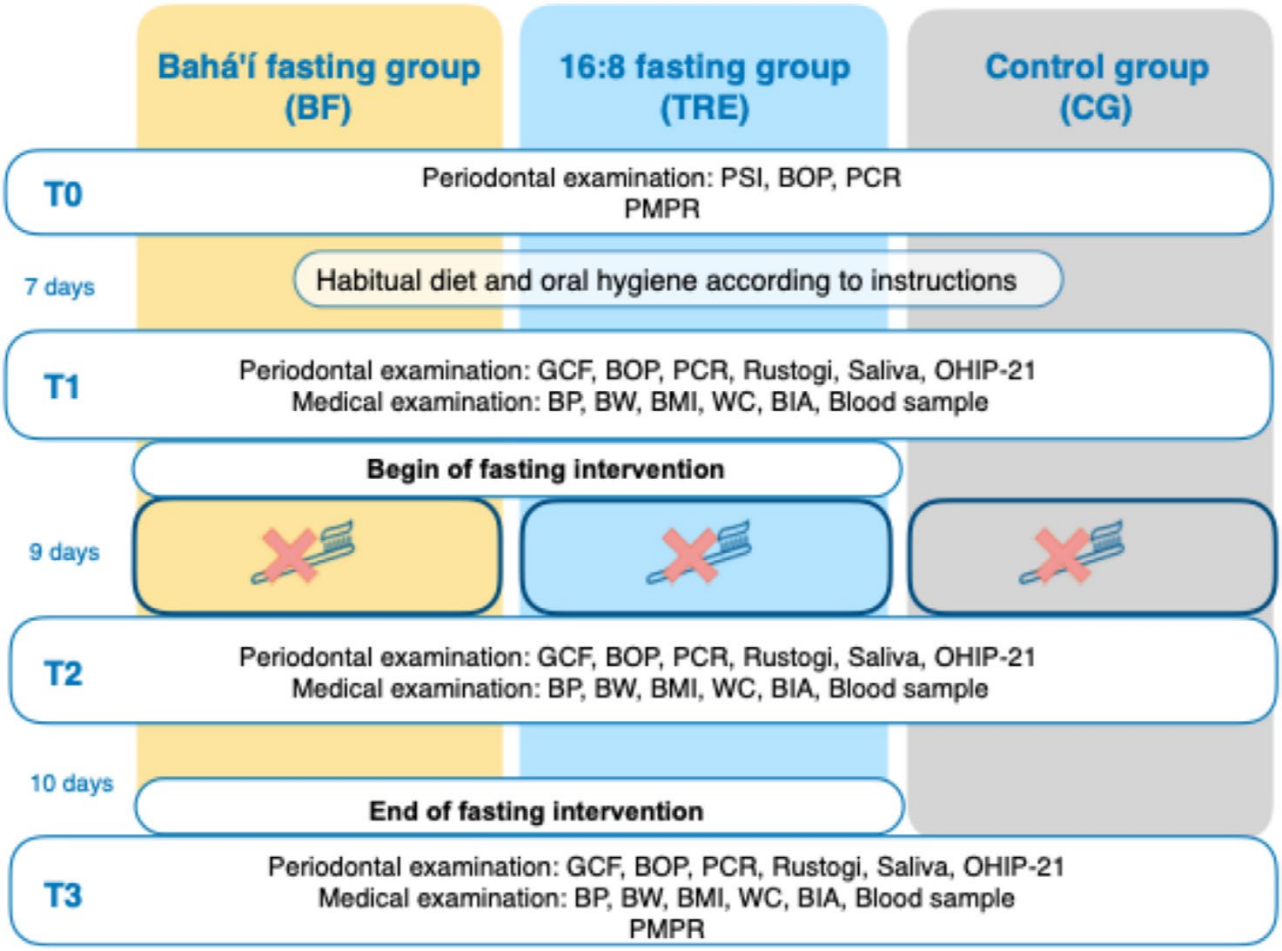
Study protocol. BIA, bioimpedance analysis; BMI, body mass index; BOP, bleeding on probing; BP, blood pressure; BW, body weight; GCF, gingival crevicular fluid; HbA1c, glycated haemoglobin; OHIP, oral health impact profile; PCR, plaque control record; PMPR, professional mechanical plaque removal; PSI, periodontal screening index.

This three‐armed clinical trial was approved by the institutional ethical review boards of the Charité—Universitätsmedizin Berlin (EA2/091/21) and Martin‐Luther‐Universität Halle‐Wittenberg (2021‐135) and registered in the German Clinical Trials Register (DRKS00026701) and adheres to the CONSORT reporting guidelines. Participants were recruited and treated at the Department of Periodontology, Oral Medicine and Oral Surgery (CharitéCentrum3, Berlin) and the Department of Prosthetic Dentistry (Universitätsmedizin, Halle). Bahá'í members were recruited through the national secretary of the community (BF). After meeting the inclusion criteria, participants provided written informed consent. Non‐Bahá'í participants received a sealed envelope from an external individual (CLP) and were randomly assigned to either TRE or controls (CG), stratified by gender and centre, with block sizes being either 2 or 4 in a random order (blockrand backage in Rstudio version, 2022.12.01) generated by an external statistician (TK). All participants were instructed to withhold any information regarding their study group until the end of the study to maintain the blinding of the investigators. Study data was managed using REDCap (Research Electronic Data Capture, version 12.1.1) hosted at Charité Universitätsmedizin Berlin. The study protocol is illustrated in Figure 1.

### 2.2 Study Population

Systemically healthy participants aged 18–69 years were enrolled in the study after meeting the following inclusion criteria at baseline (T1):

- Inconspicuous dental status without caries/carious lesions and prosthetics in the test sextant with *n* ≥ 24 teeth.
- Periodontally healthy without clinical attachment loss (CAL), or on a reduced periodontium in a non‐periodontitis patient with buccal or oral CAL < 3 mm, with periodontal probing depths (PPD) ≤ 3 mm, BOP ≤ 10% (applied for baseline T1, not pre‐screening T0).
- Member of the Bahá'í community planning to fast (for BF only).

The following exclusion criteria were applied:

- Periodontitis (interdental CAL on ≥ 2 non‐adjacent teeth, or buccal or oral CAL ≥ 3 mm with PPD > 3 mm detectable at ≥ 2 teeth) reduces periodontium in a stable periodontitis patient (Chapple et al. 2018; Tonetti et al. 2018).
- PPD > 3 mm, BOP > 10% (applied for baseline T1, not pre‐screening T0).
- Untreated caries or carious lesions, even if only preventively controlled.
- Orthodontic appliances, ongoing dental treatment, xerostomia, mucogingival disorders.
- Pregnancy and lactation.
- Use of antibiotics in the past 3 months.
- Any medication interfering with periodontal bleeding or causing gingival overgrowth (e.g., antiphlogistic medication, calcium channel blockers).
- History of eating disorders (anorexia nervosa, bulimia).
- Severe psychiatric or medical illness.
- Participation in another study.
- Smoking > 5 cigarettes/day.
- Planned interruption of religious fasting for more than one day during T1–T2 (BF only).

### 2.3 Pre‐Treatment and Experimental Gingivitis Model

The screening and pre‐treatment were scheduled one week prior to the study beginning (T0) and included the assessment of medical history, dental status/DMFT index (Klein et al. 1938), periodontal screening index (Meyle and Jepsen 2000) as well as a full mouth bleeding on probing (BOP) (Ainamo and Bay 1975) and plaque control record (PCR) (O'Leary et al. 1972). A subsequent professional mechanical plaque removal (PMPR) including oral hygiene instructions was performed to reduce gingival inflammation prior to the study. Baseline measurements (T1) and final inclusion were performed on the day when the experimental gingivitis phase (test sextant teeth 24–27) started, which lasted for 9 days (T2) exceeding the originally planned timepoints due to participants' availability (Figure 1). If crowns or prosthetics were present in that area, the opposite upper side was determined (teeth 14–17). A large red adhesive sticker was provided as a reminder on bathroom mirrors. Additionally, all participants received the same toothpaste (elmex Kariesschutz professional Zahnpasta, CP GABA GmbH, Germany) containing no antiseptic agents and were advised not to use additional mouth rinse, chewing gum or to chew hard‐textured foods on the test side during T1–T2 (for more details Appendix S1 and Figure 1).

### 2.4 Fasting Interventions

Bahá'í abstain from consuming drink and food from sunrise until sunset. This fast is performed annually in March for 19 days, coinciding with the month when day and night durations are approximately equal (equinox) leading to a daytime fast of around 12 h. Food intake is typically reduced to early in the morning and evenings (Koppold‐Liebscher et al. 2021). Participants in the TRE group were permitted to consume isocaloric solid foods and beverages within an eight‐hour window during the day, which they were free to choose. Outside of this window, only non‐caloric beverages such as water or tea were allowed (Schuppelius et al. 2021). Fasting interventions started after T1 and ended after 19 days (T3). The CG was told to continue with their habitual eating window. All participants were instructed not to alter the types of foods they typically consume during the study period and were asked to document their eating and fasting hours (T1–T3) (for more details Appendix S1 and Figure 1).

### 2.5 Oral and Clinical Examination

Two blinded and experienced examiners in Berlin (JM) and Halle (SD) assessed the following parameters for all groups at all time points (T1–T3): full mouth BOP using a pressure‐calibrated probe (periodontometer, UNC15, Aesculap AG, Germany) and PCR using a fluorescent staining solution (Plaque Test indicator liquid, Ivoclar Vivadent GmbH, Germany). Additionally, the Rustogi plaque index (RPI) was measured (Rustogi et al. 1992) and a BOP_s was separately calculated, both for the test sextant. All parameters were calculated in %. GCF was assessed using the Periotron 8000 device (Berlin) and Periotron 6000 (Halle) (Oraflow Inc., USA). Four samples were collected for 30 s from both interproximal areas of the upper tested premolars under dry and cleaned conditions using PerioPaper (Oralflow Inc., USA). Mean values were calculated from the repeated measurements and transferred into microliters using a standard curve. Stimulated saliva volume was collected for 3 min and pH levels were determined using a calibrated scale (MK‐2000B Petite Balance, YMC, Japan) and pH test strips (pH‐Fix 0–14, Carl Roth GmbH + Co. KG; Germany), respectively. Metabolic parameters were assessed at T1–T3. Oral health‐related quality of life was assessed using the Oral Health Impact Profile (OHIP‐21) (for more details and additional inquiries see Appendix S1 and Figure 1).

Differences in BOP_s between groups at T2 (with T1 values as covariates in the ANCOVAs) were considered the primary outcome, while other parameter changes were secondary outcomes.

### 2.6 Statistical Analyses

Descriptive statistics comprise absolute and relative frequencies for categorical variables and mean and standard deviation (SD) for continuous variables. Oral parameters (BOP, BOP_s, PCR, RPI) are presented as mean percentages and averaged across all patients per group.

Sample size calculation was based on the assumption of a BOP of 25% ± 10 after 9 days in the CG and a group difference between CG and the other groups of 10% (Dommisch et al. 2019; 2015; Pappe et al. 2021) resulting in an estimated effect size of *f* = 0.39, corresponding to a Cohen's *d* = 0.78. Based on using an ANOVA and the standard parameter (alpha = 0.05 and beta = 0.20) it resulted in an optimal *n* = 18 participants or 21 including an estimated drop‐out of 15% in each group.

Only the primary endpoint was tested on a confirmatory basis using this alpha of 0.025. All other comparisons were conducted on an exploratory level, without any correction for multiple testing, and tests with *p* < 0.05 are thus considered interesting for future studies but are not taken as confirmatory evidence. Among these secondary analyses were the following: (1) The ANCOVA for the main parameter (BOP_s) was extended for a subgroup of individual covariates to test their impact on the primary results. (2) Mean differences between time points, along with confidence interval (CI) were reported and tested using dependent‐samples *t*‐tests to assess intragroup changes between T1 and T2 or T3, respectively. (3) Finally, all data were re‐analysed using mixed ANOVA with within factor timepoints and in‐between factor groups. There, next to standard *p*‐values, corrected *p*‐values are given for cases in which data were not normally distributed (Tables S5 and S6). Analyses were conducted using custom‐written code in Python (version 3.9, with packages statsmodels and scipy). Prior single imputation of the individual missing values for T3 was done using the single imputer of SciKitLearn (version 1.2).

## 3 Results

### 3.1 Population and Drop‐Out Analyses

**FIGURE 2:**
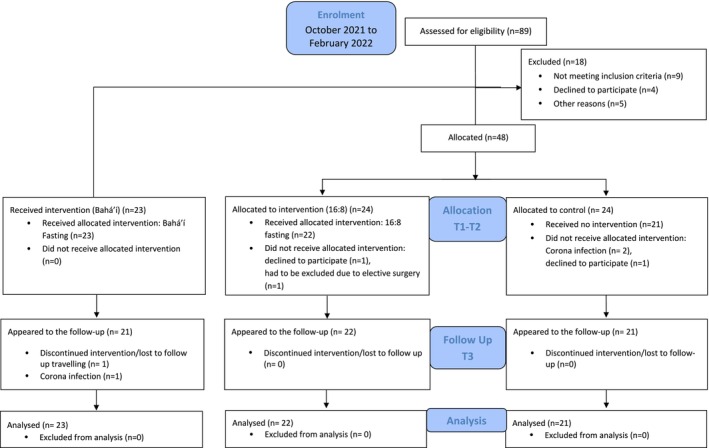
CONSORT flow diagram of the number of included participants and after correction for excluded participants and dropouts.

Between October 2021 and February 2022, 89 individuals were screened, of whom 9 were ineligible and 4 declined to participate. Five participants dropped out for reasons unrelated to the study. The study measurements were taken between January and June 2022. Forty‐eight participants were allocated to either the TRE or the CG. During the study, two controls had to withdraw due to coronavirus infection, and one declined to continue. In the TRE, one participant declined to continue, and one was excluded due to elective extraoral surgery. Ultimately, 22 participants from the TRE and 21 controls received the intervention (T1–T2) and appeared for the follow‐up (T3). Additionally, 23 Bahá'í were included in receiving the intervention until T2. One Bahá'í contracted COVID‐19, and another discontinued fasting due to travel commitments. In total, data from 23 participants were analysed, with missing data for T3 imputed (Figure 2).

Mean age was 32.04 (11.82) years in BF, 30.09 (5.79) years in TRE and 29.1 (4.26) years in CG. Differences between groups were observed in GCF, waist circumference (WC), triglycerides (TRG) and high‐density lipoprotein (HDL). Sociodemographic, metabolic and periodontal parameters at baseline are summarised in Table 1.

**TABLE 1:** Baseline values (T1) characterising the study population.

T1	Bahá'í fasting group (BF)	16:8 fasting group (TRE)	Control group (CG)	*p*‐*χ* ^2^‐value
	*n* (%)	*n* (%)	*n* (%)	
Total	23 (100)	22 (100)	21 (100)	
Clinic_Berlin	11 (47.8)	14 (63.6)	14 (66.7)	0.431
Clinic_Halle	12 (52.2)	8 (36.4)	7 (33.3)	
Female	11 (47.8)	16 (72.7)	14 (66.7)	0.311
Diet				
Omnivor	13 (56.5)	8 (36.4)	6 (28.6)	0.885^a^
Pescetarian	0 (0)	2 (9.1)	2 (9.5)	
Lacto‐ovo veg.	5 (21.7)	7 (31.8)	8 (38.1)	
Lacto‐veg.	1 (4.3)	1 (4.5)	0 (0)	
Vegan	2 (8.7)	2 (9.1)	3 (14.3)	
Other	2 (8.7)	2 (9.1)	2 (9.5)	
Non smokers	23 (100)	22 (100)	21 (100)	0.999
Test sextant 3	21 (91.3)	20 (95.2)	16 (76.2)	0.138

	Mean (SD)	Mean(SD)	Mean (SD)	*p*‐ANOVA	BF vs. TRE	TRE vs. CG	BF vs. CG
Age	32.04 (11.82)	30.09 (5.79)	29.1 (4.26)	0.488			
BOP (%)	4.85 (2.79)	5.43 (3.48)	5.77 (2.89)	0.619			
GCF (μL)	0.25 (0.14)	0.15 (0.12)	0.14 (0.13)	**0.019**	**0.023**	0.734	0.016
PCR (%)	17.11 (7.93)	15.52 (8.39)	13.54 (6.69)	0.332			
Saliva (g/min)	1.52 (0.85)	1.87 (1.05)	1.59 (0.90)	0.453			
pH saliva	7.26 (0.53)	7.25 (0.82)	7.4 (0.68)	0.724			
OHIP‐21	5.7 (6.13)	3.59 (3.88)	4.43 (5.52)	0.424			
DMFT	4 (3.86)	3.23 (3.88)	2.19 (2.77)	0.264			
BW (kg)	78.2 (15.2)	69.9 (12.6)	72.1 (15.14)	0.154			
BMI	25.22 (4.15)	23.72 (3.39)	23.75 (3.87)	0.342			
WC (cm)	87.07 (13.44)	79.77 (7.95)	81.81 (11.03)	**0.091**	**0.036**	0.503	0.172
WHR	0.92 (0.06)	0.89 (0.05)	0.9 (0.08)	0.284			
BP_sys (mmHg)	129.87 (11.73)	121.68 (11.4)	125.43 (15.28)	0.121			
BP_dia (mmHg)	82.35 (11.3)	80.18 (8.76)	77.67 (10.9)	0.351			
FATv (%)	6.43 (3.98)	4.86 (1.77)	5.38 (2.9)	0.232			
FAT (%)	28.47 (11.67)	27.65 (7.46)	27.36 (7.06)	0.917			
MM (%)	32.78 (7.82)	32.23 (4.55)	31.79 (6.5)	0.882			
CHOL (mg/dL)	182.61 (48.78)	167.82 (30.44)	169.52 (24.69)	0.354			
TRG (mg/dL)	140.7 (125.37)	82.64 (47.08)	88 (44.46)	**0.046**	0.052	0.709	0.075
HDL (mg/dL)	46.61 (12.1)	55.59 (13.2)	56.81 (13.21)	**0.023**	**0.025**	0.769	**0.013**
LDL (mg/dL)	106.04 (37.87)	90.5 (25.41)	90.86 (21.96)	0.151			
CRP (mg/L)	1.49 (1.22)	0.88 (0.73)	1.13 (1.33)	0.213			
IL‐6 (ng/L)	2.03 (1.26)	1.59 (0.21)	1.76 (0.75)	0.257			
HbA1c (%)	5.22 (0.36)	5.15 (0.21)	5.1 (0.24)	0.366			

*Note*: Bold values indicates statistical significance *p* < 0.05.

Abbreviations: BIA, bioimpedance analysis; BMI, body mass index; BOP, bleeding on probing; BP_dia, diastolic blood pressure; BP_sys, systolic blood pressure; BW, body weight; CHOL, total cholesterol; CRP, C‐reactive protein; d, day; DMFT, decayed missing filled teeth; FAT, total fat mass; FATv, visceral fat mass; GCF, gingival crevicular fluid; HbA1c, glycated haemoglobin; HDL, high density lipoprotein; IL, interleukin; LDL, low density lipoprotein; MM, muscle mass; *n*, number, OHIP, oral health impact profile; PCR, plaque control record; PISA, periodontal inflamed surface area; SD, standard deviation; TRG, triglycerides; WC, waist circumference; WHR, waist to hip ratio.

^a^ The *p*‐value refers to a *χ* ^2^‐test assessing the difference in the distributions across all diets.

### 3.2 Fasting Intervention

Self‐reported fasting duration overnight increased significantly in the TRE by 3.55 h (4.27) to 14.86 h (3.4) after one week and remained at a mean of 14.55 h (3.34) at T3 (intra‐*p*‐value_TRE_ T1–T2 = 0.001, T1–T3 < 0.001). Total fasting time (night and day) calculated for BF increased significantly from 11.04 h (2.77) to 16.16 h (4.58) at T2 and remained high at a mean of 16.48 h (4.32) (intra‐*p*‐value_BF_ T1–T2 = 0.0001, T1–T3 < 0.0001).

The CG remained at a mean fasting time of 11 h throughout the study and differed significantly from the fasting groups (Table S1). Data from continuous glucose monitoring of a sub‐group indicates a significant calorie reduction in the BF only (BF: −678 kcal; TRE: −275 kcal, CG: −51 kcal); however, the macronutrient composition remained unchanged in all groups (Peters et al. 2024).

### 3.3 Effects of Intermittent Fasting on Oral Parameters

Intra‐group comparisons between T1 and T2 showed significant increases in BOP_s: 14.61% (11.13) in the TRE, 14.9% (15.26) in the CG and 5.42% (7.4) in the BF group. This resulted in significant inter‐group differences in change between BF and CG (−9.48% [−17.18; −1.79]) and BF and TRE (−9.19% [−15.07; −3.32]) with lower BOP values in the BF.

GCF significantly increased from T1 to T2 by 0.09 μL (0.13) within the CG but remained unchanged in the fasting groups, resulting in significant inter‐group differences in change between BF and CG (−0.06 μL [−7.22; −0.66]) and TRE and CG (−0.08 [−0.17; −0.00]) (Table 2). Both plaque indices increased significantly within all groups (intra‐*p*‐value < 0.001; Table 2).

**TABLE 2:** Periodontal parameters are given for both fasting groups and controls (only T1 and T2 in parentheses).

Periodontal parameters		*n*	T1	T2	T2 − T1			TRE‐CG	BF‐CG	BF‐TRE
								ΔT2 − T1 [CI]		
			Mean (SD)	Mean (SD)	Diff.	[CI]	Intra‐*p* value^a^	Inter‐*p* value^b^		
BOP_s (%)	TRE	22	8.47 (6.93)	23.08 (12.78)	14.61	[9.85; 19.37]	**< 0.001**	−0.29	−9.48	−9.19
	BF	23	4.71 (4.48)	10.13 (7.65)	5.42	[2.32; 8.51]	**0.002**	[−8.78; 8.20]	[−17.18; −1.79]	[−15.07; −3.32]
	CG	21	7.11 (5.15)	22 (17.36)	14.9	[8.21; 21.58]	**< 0.001**	0.946	**0.018**	**0.003**
BOP (%)	TRE	22	5.43 (3.48)	12.09 (10.76)	6.66	[2.23; 11.08]	**0.008**	1.9	−3.94	−5.84
	BF	23	4.85 (2.79)	5.67 (4.34)	0.82	[−0.62; 2.25]	0.279	[−3.53; 7.32]	[−7.22; −0.66]	[−10.73; −0.96]
	CG	21	5.77 (2.89)	10.53 (7.6)	4.76	[1.96; 7.56]	**0.003**	0.482	**0.02**	**0.021**
GCF (μL)	TRE	22	0.15 (0.12)	0.16 (0.14)	0.02	[−0.05; 0.06]	0.877	−0.08	−0.06	0.02
	BF	23	0.24 (0.14)	0.27 (0.12)	0.03	[−0.06; 0.11]	0.539	[−0.17; −0.00]	[−0.16; 0.04]	[−0.08; 0.13]
	CG	21	0.14 (0.13)	0.23 (0.13)	0.09	[0.03; 0.14]	**0.006**	**0.049**	**0.026**	0.674
RPI (%)	TRE	22	16.47 (15.16)	77.94 (13.47)	61.46	[54.20; 68.73]	**< 0.001**	0.95	1.62	0.67
	BF	23	16.21 (11.29)	78.35 (11.26)	62.14	[56.22; 68.05]	**< 0.001**	[−11.27; 13.17]	[−9.83; 13.08]	[−8.98; 10.33]
	CG	21	15.08 (12.78)	75.6 (19.8)	60.52	[51.18; 69.85]	**< 0.001**	0.876	0.775	0.889
PCR (%)	TRE	22	15.52 (8.39)	34.03 (15.6)	18.51	[11.75; 25.27]	**< 0.001**	2.9	3.9	1
	BF	23	17.11 (7.93)	36.19 (11.09)	19.08	[12.34; 26.67]	**< 0.001**	[−5.87; 11.68]	[−5.21; 13.01]	[−9.14; 11.14]
	CG	21	13.54 (6.69)	29.14 (12.89)	15.61	[10.45; 20.76]	**< 0.001**	0.507	0.482	0.843
Saliva (g/min)	TRE	22	1.87 (1.05)	1.78 (0.88)	−0.084	[−0.41; 0.25]	0.625	−360.58	−43.41	317.17
	BF	23	1.52 (0.85)	1.76 (0.65)	0.23	[−0.051; 0.52]	0.122	[−769.91; 48.75]	[−411.18; 324.35]	[−132.61; 766.94]
	CG	21	1.59 (0.90)	1.86 (0.97)	0.28	[0.061; 0.49]	**0.020**	0.082	0.861	0.162
pH	TRE	22	7.25 (0.82)	7.25 (0.7)	0	[−0.17; 0.17]	1.000	0.07	0.27	0.2
	BF	23	7.36 (0.53)	7.46 (0.74)	0.2	[−0.04; 0.43]	0.119	[−0.23; 0.37]	[−0.08; 0.61]	[−0.11; 0.50]
	CG	21	7.4 (0.68)	7.33 (0.56)	−0.07	[−0.31; 0.17]	0.561	0.635	0.126	0.196
OHIP‐21	TRE	22	3.59 (3.88)	5.82 (4.52)	2.23	[−0.24; 4.69]	0.091	2.32	−0.51	−2.84
	BF	23	5.7 (6.13)	5.09 (5.8)	−0.61	[−3.93; 2.72]	0.723	[−1.45; 6.10]	[−4.93; 3.90]	[−7.10; 1.43]
	CG	21	4.43 (5.52)	4.33 (4.59)	−0.1	[−2.80; 2.61]	0.946	0.221	0.816	0.187

*Note*: Bold values denote statistical significance at the *p* < 0.05 level.

Abbreviations: BF, Bahá'í fasting group; BOP, bleeding on probing; BOP_s, bleeding on probing in test sextant (24–27); CG, control group; CI, confidence interval; GCF, gingival crevicular fluid; OHIP, oral health impact profile; PCR, plaque control record; RPI, Rustogi plaque index; SD, standard deviation; TRE, time‐restricted eating/16:8 fasting group.

^a^ Paired *t*‐test.

^b^ *t*‐test.

Influencing factors were examined in a separate ANCOVA analysis. Significant co‐factors related to the outcome of BOP_s (difference T2 − T1) were dental centre (*p* < 0.001) in all group comparisons (BF‐TRE, BF‐CG, TRE‐CG) and PCR for BF–TRE (*p* < 0.001) and TRE–CG (*p* < 0.05). When analysing data separately by the centre, the treatment effect for BOP_s remained stable for the BF (Berlin) (inter‐*p*‐value_CG‐BF_ = 0.048, *p*_TRE‐BF_ = 0.006) (Table S2).

At T3 after oral hygiene was resumed, GCF returned to similar levels relative to T1 in the CG. GCF levels remained unchanged throughout all time points within the TRE, whereas a significant reduction was shown in the BF (intra‐*p*‐value = 0.003). GCF inter‐group comparisons revealed significant differences in change between BF and CG (−0.09 μL [−0.18; −0.01]).

Within each group, BOP_s decreased compared with T2 but remained higher, differing significantly from T1 values. Inter‐group comparisons showed no significant differences (Table 3). A separate analysis of BOP revealed that the values were similar to T0 prior to PMPR (Table S3).

**TABLE 3:** Periodontal parameters are given for both fasting groups and controls (only T1 and T3 in parentheses).

Periodontal parameters		*n*	T1	T3	T3 − T1			TRE‐CG	BF‐CG	BF‐TRE
								ΔT3 − T1 [CI]		
			Mean (SD)	Mean (SD)	Diff.	[CI]	Intra‐*p* value^a^	Inter‐*p* value^b^		
BOP_s (%)	TRE	22	8.47 (6.93)	14.55 (9.64)	6.08	[2.78; 9.38]	**0.002**	−0.67	−0.45	0.22
	BF	23	4.71 (4.48)	10.85 (7.55)	6.13	[2.98; 9.62]	**0.002**	[−6.37; 5.04]	[−6.17; 5.27]	[−4.60; 5.03]
	CG	21	7.11 (5.15)	13.86 (11.39)	6.75	[2.32; 11.18]	**0.007**	0.814	0.831	0.928
BOP (%)	TRE	22	5.43 (3.48)	11.31 (8.98)	5.88	[2.16; 9.60]	**0.005**	2.82	−1.21	−4.02
	BF	23	4.85 (2.79)	6.66 (4.3)	1.81	[0.30; 3.42]	**0.032**	[−1.52; 7.16]	[−3.73; 1.32]	[−8.24; 0.19]
	CG	21	5.77 (2.89)	8.83 (5.71)	3.06	[1.18; 4.95]	**0.005**	0.195	0.322	0.061
GCF (μL)	TRE	22	0.15 (0.12)	0.12 (0.11)	−0.04	[−0.09; 0.02]	0.186	−0.02	−0.09	−0.08
	BF	23	0.24 (0.14)	0.13 (0.1)	−0.11	[−0.18; −0.05]	**0.003**	[−0.09; 0.06]	[−0.18; −0.01]	[−0.16; 0.00]
	CG	21	0.14 (0.13)	0.12 (0.14)	−0.02	[−0.07; 0.03]	0.427	0.676	**0.026**	0.064
RPI (%)	TRE	22	16.47 (15.16)	26.7 (14.7)	10.23	[2.61; 17.85]	**0.016**	2.19	3.29	1.09
	BF	23	16.21 (11.29)	26.54 (12.84)	10.32	[4.27; 18.38]	**0.008**	[−7.56; 11.95]	[−5.98; 12.55]	[−9.59; 11.78]
	CG	21	15.08 (12.78)	23.11 (13.1)	8.03	[2.46; 13.61]	**0.010**	0.651	0.617	0.837
PCR (%)	TRE	22	15.52 (8.39)	27.42 (18.18)	11.9	[4.82; 18.99]	**0.003**	0.88	2	1.12
	BF	23	17.11 (7.93)	29.01 (19.08)	11.9	[7.61; 18.43]	**< 0.001**	[−8.71; 10.47]	[−6.35; 10.35]	[−8.07; 10.31]
	CG	21	13.54 (6.69)	24.56 (15.77)	11.02	[4.99; 17.05]	**0.002**	0.854	0.828	0.806
Saliva (g/min)	TRE	22	1.87 (1.05)	1.78 (1.02)	−0.09	[−0.40; 0.22]	0.569	−97.65	30.33	127.98
	BF	23	1.52 (0.85)	1.56 (0.78)	0.037	[−0.19; 0.26]	0.445	[−466.21; 270.92]	[−265.35; 326.01]	[−263.72; 519.67]
	CG	21	1.59 (0.90)	1.59 (0.79)	0.007	[−0.17; 0.19]	0.938	0.594	0.589	0.513
pH	TRE	22	7.25 (0.82)	7.25 (0.79)	0	[−0.11; 0.11]	1.000	−0.1	0.04	0.13
	BF	23	7.36 (0.53)	7.39 (0.81)	0.13	[−0.12; 0.38]	0.314	[−0.32; 0.13]	[−0.28; 0.36]	[−0.15; 0.41]
	CG	21	7.4 (0.68)	7.5 (0.69)	0.1	[−0.09; 0.28]	0.329	0.397	0.825	0.355
OHIP‐21	TRE	22	3.59 (3.88)	4.36 (2.85)	0.77	[−0.98; 2.53]	0.398	1.2	−2.83	−4.03
	BF	23	5.7 (6.13)	2.43 (2.1)	−3.26	[−5.91; −0.61]	**0.024**	[−1.70; 4.10]	[−6.37; 0.71]	[−7.31; −0.75]
	CG	21	4.43 (5.52)	4 (2.16)	−0.43	[−2.62; 1.77]	0.706	0.407	0.114	**0.017**

*Note*: Bold values denote statistical significance at the *p* < 0.05 level.

Abbreviations: BF, Bahá'í fasting group; BOP, bleeding on probing; BOP_s, bleeding on probing in test sextant (24–27); CG, control group; CI, confidence interval; GCF, gingival crevicular fluid; OHIP, oral health impact profile; PCR, plaque control record; RPI, Rustogi plaque index; SD, standard deviation; TRE, time‐restricted eating/16:8 fasting group.

^a^ Paired *t*‐test.

^b^ *t*‐test.

PCR had a significant effect (*p* < 0.05) on the outcome of BOP_s (difference T3 − T1) in BF compared with TRE. The centre effect was significant for GCF (difference T3 − T1) when comparing BF–TRE (< 0.01) and BF–CG (< 0.05). Dietary pattern, sugar intake and fasting time showed no influence on the results (T2 − T1, T3 − T1).

### 3.4 Effects of Intermittent Fasting on Metabolic Parameters

Within the BF, body weight significantly decreased at T2 by 1.08 kg (1.04) compared with T1, with inter‐group comparisons revealing a significant difference from the other groups. Further, significant intra‐group reductions (T2 − T1) were observed for blood pressure (BP), C‐reactive protein (CRP) and triglycerides in the BF (Table 4). Intra‐group comparisons for T3 − T1 revealed significant reductions in body weight and BMI in both fasting groups and a significant decrease in systolic BP, total fat and lipids within the BF only. HbA1c levels decreased from T1 to T3 significantly within both fasting groups (Table 5).

**TABLE 4:** Metabolic parameters are given for both fasting groups and controls (only T1 and T2 in parentheses).

Metabolic parameters		*n*	T1	T2	T2 − T1			TRE‐CG	BF‐CG	BF‐TRE
								ΔT2 − T1 [CI]		
			Mean (SD)	Mean (SD)	Diff.	[CI]	Intra‐*p* value^a^	Inter‐*p* value^b^		
BW (kg)	TRE	22	69.9 (12.6)	69.63 (12.3)	−0.28	[−0.73; 0.18]	0.247	0.03	−0.77	−0.81
	BF	23	78.2 (15.2)	77.11 (15.1)	−1.08	[−1.52; −0.65]	**< 0.001**	[−0.62; 0.69]	[−1.41; −0.13]	[−1.45; −0.16]
	CG	21	72.1 (15.14)	71.79 (15.16)	−0.31	[−0.75; 0.13]	0.185	0.921	**0.019**	**0.016**
BMI	TRE	22	23.72 (3.39)	23.64 (3.33)	−0.08	[−0.23; 0.07]	0.301	0.03	−0.25	−0.28
	BF	23	25.22 (4.15)	4.15 (24.86)	−0.36	[−0.50; −0.22]	**< 0.001**	[−0.19; 0.24]	[−0.46; −0.05]	[−0.49; −0.07]
	CG	21	23.75 (3.87)	23.64 (3.87)	−0.11	[−0.25; 0.04]	0.156	0.807	**0.016**	**0.01**
BP_sys (mmHg)	TRE	22	121.68 (11.4)	126.5 (12.45)	4.82	[−0.05; 9.68]	0.066	4.63	−6.02	−10.64
	BF	23	129.87 (11.73)	124.04 (12.61)	−5.83	[−9.30; −2.35]	**0.003**	[−2.38; 11.63]	[−12.10; 0.07]	[−16.82; −4.47]
	CG	21	125.43 (15.28)	125.62 (16.98)	0.19	[−4.56; 4.94]	0.938	0.190	0.052	**0.001**
FAT_v (%)	TRE	22	4.86 (1.77)	4.86 (1.55)	0	[−0.32; 0.32]	1.000	0.24	0.19	−0.04
	BF	23	6.43 (3.98)	6.39 (3.75)	−0.04	[−0.30; 0.22]	0.747	[−0.14; 0.62]	[−0.14; 0.53]	[−0.47; 0.38]
	CG	21	5.38 (2.9)	5.14 (3.01)	−0.24	[−0.42; −0.05]	**0.021**	0.212	0.241	0.836
TRG (mg/dL)	TRE	22	82.64 (47.08)	75.55 (38.23)	−7.09	[−21.02; 6.84]	0.330	−6.47	−48.29	−41.82
	BF	23	142.65 (124.71)	88.52 (42.78)	−54.1	[−97.16; −0.67]	**0.031**	[−31.47; 18.52]	[−102.68; 6.09]	[−94.52; 10.88]
	CG	21	88 (44.46)	87.38 (47.45)	−0.62	[−20.36; 19.12]	0.952	0.603	**0.048**	0.115
HDL (mg/dL)	TRE	22	55.59 (13.2)	59.27 (14.84)	3.68	[0.55; 6.82]	**0.032**	5.4	0.98	−4.42
	BF	23	46.22 (11.68)	46.09 (10.59)	−0.13	[−3.23; 1.75]	0.912	[0.82; 9.97]	[−3.16; 5.11]	[−8.55; −0.29]
	CG	21	56.81 (13.21)	55.1 (10.9)	−1.71	[−4.86; 1.43]	0.298	**0.022**	0.428	**0.036**
CRP (mg/L)	TRE	22	0.88 (0.73)	0.79 (0.45)	−0.1	[−0.36; 0.17]	0.487	0.2	−0.04	−0.24
	BF	23	1.49 (1.22)	1.11 (1)	−0.37	[−0.69; 0.01]	**0.047**	[−0.47; 0.87]	[−0.75; 0.67]	[−0.70; 0.21]
	CG	21	1.13 (1.33)	0.83 (0.4)	−0.3	[−0.88; 0.28]	0.326	0.537	0.833	0.283
HbA1c (%)	TRE	22	5.15 (0.21)	5.11 (0.23)	−0.04	[−0.12; 0.04]	0.329	−0.03	−0.01	0.02
	BF	23	5.23 (0.36)	5.2 (0.39)	−0.03	[−0.07; 0.03]	0.236	[−0.13; 0.07]	[−0.09; 0.07]	[−0.08; 0.12]
	CG	21	5.1 (0.24)	5.09 (0.23)	−0.01	[−0.07; 0.04]	0.624	0.597	0.615	0.695

*Note*: Bold values denote statistical significance at the *p* < 0.05 level.

Abbreviations: BF, Bahá'í fasting group; BMI, body mass index; BP_sys, systolic blood pressure; BW, body weight; CG, control group; CI, confidence interval; CRP, C‐reactive protein; FAT_v, visceral fat; HbA1c, glycated haemoglobin; HDL, high‐density lipoprotein; SD, standard deviation; TRE, time‐restricted eating/16:8 fasting group; TRG, triglycerides.

^a^ Paired *t*‐test.

^b^ *t*‐test.

**TABLE 5:** Metabolic parameters are given for both fasting groups and controls (only T1 and T3 in parentheses).

Metabolic parameters		T1	T3	T3 − T1			TRE‐CG	BF‐CG	BF‐TRE
							ΔT3 − T1 [CI]		
		Mean (SD)	Mean (SD)	Diff.	[CI]	Intra‐*p* value^a^	Inter‐*p* value^b^		
BW (kg)	TRE	69.9 (12.6)	69.33 (12.24)	−0.58	[−1.09; −0.06]	**0.040**	−0.28	−2.6	−2.32
	BF	78.2 (15.2)	75.45 (13.45)	−2.74	[−5.63; −0.17]	**0.046**	[−1.04; 0.48]	[−5.53; 0.33]	[−5.25; 0.61]
	CG	72.1 (15.14)	71.8 (15.22)	−0.3	[−0.82; 0.22]	0.276	0.465	0.078	0.115
BMI	TRE	23.72 (3.39)	23.52 (3.26)	−0.19	[−0.35; −0.03]	**0.029**	−0.09	−0.99	−0.9
	BF	25.22 (4.15)	24.11 (4.06)	−1.11	[−1.95; −0.24]	**0.022**	[−0.33; 0.15]	[−1.91; −0.07]	[−1.82; 0.01]
	CG	23.75 (3.87)	23.64 (3.89)	−0.1	[−0.27; 0.06]	0.238	0.464	**0.039**	**0.053**
BP_sys (mmHg)	TRE	121.68 (11.4)	122.36 (11.11)	0.68	[−3.02; 4.38]	0.721	2.92	−5.11	−8.03
	BF	129.87 (11.73)	123.96 (14.38)	−5.91	[−11.06; −3.63]	**0.024**	[−3.94; 9.78]	[−11.98; 1.76]	[−13.42; −2.64]
	CG	125.43 (15.28)	123.19 (14.16)	−2.24	[−7.73; 3.26]	0.434	0.393	0.328	**0.004**
FAT (%)	TRE	27.65 (7.46)	27.18 (8.13)	−0.47	[−1.56; 0.63]	0.411	0.17	−2.68	−2.85
	BF	28.47 (11.67)	25.13 (10.14)	−3.34	[−5.85; −0.79]	**0.020**	[−1.23; 1.57]	[−5.46; 0.10]	[−5.73; 0.02]
	CG	27.36 (7.06)	26.72 (6.63)	−0.64	[−1.43; 0.16]	0.132	0.807	0.063	**0.051**
CHOL (mg/dL)	TRE	167.82 (30.44)	172.18 (27.46)	4.36	[−2.54; 11.27]	0.229	15.65	−5.24	−20.89
	BF	182.96 (48.71)	165.52 (27.83)	−17.43	[−28.68; −4.36]	**0.010**	[4.61; 26.69]	[−20.37; 9.90]	[−35.37; −6.40]
	CG	169.52 (24.69)	158.24 (25.09)	−11.29	[−19.47; −3.11]	**0.014**	**0.007**	0.476	**0.006**
TRG (mg/dL)	TRE	82.64 (47.08)	85.36 (102.74)	2.73	[−32.30; 37.75]	0.880	5.06	−28.54	−33.6
	BF	142.65 (124.71)	93.7 (51.06)	−48.96	[−86.42; 24.68]	**0.033**	[−34.17; 44.29]	[−88.62; 31.55]	[−101.49; 34.30]
	CG	88 (44.46)	85.67 (36.02)	−2.33	[−15.63; 10.96]	0.734	0.793	**0.048**	0.323
LDL (mg/dL)	TRE	90.5 (25.41)	92.09 (21.4)	1.59	[−4.87; 8.05]	0.634	11.020	−1.05	−12.07
	BF	106.52 (37.95)	95.26 (27.28)	−11.26	[−18.28; −2.68]	**0.009**	[1.39; 20.65]	[−11.68; 9.58]	[−22.50; −1.64]
	CG	90.86 (21.96)	81.43 (19.3)	−9.43	[−16.19; −2.67]	**0.013**	**0.026**	0.789	**0.024**
CRP (mg/L)	TRE	0.88 (0.73)	0.75 (0.35)	−0.13	[−0.39; 0.12]	0.325	0.16	−0.21	−0.38
	BF	1.49 (1.22)	0.98 (0.73)	−0.51	[−0.94; −0.07]	**0.035**	[−0.51; 0.84]	[−0.97; 0.54]	[−0.90; 0.15]
	CG	1.13 (1.33)	0.83 (0.52)	−0.3	[−0.89; 0.30]	0.339	0.623	0.575	0.152
HbA1c (%)	TRE	5.15 (0.21)	5.1 (0.21)	−0.05	[−0.09; −0.00]	**0.047**	−0.02	−0.06	−0.04
	BF	5.23 (0.36)	5.12 (0.31)	−0.11	[−0.15; −0.02]	**0.010**	[−0.09; 0.05]	[−0.14; 0.03]	[−0.12; 0.04]
	CG	5.1 (0.24)	5.07 (0.25)	−0.03	[−0.08; 0.02]	0.284	0.620	0.103	0.291

*Note*: Bold values denote statistical significance at the *p* < 0.05 level.

Abbreviations: BF, Bahá'í fasting group; BMI, body mass index; BP_sys, systolic blood pressure; BW, body weight; CG, control group; CHOL, total cholesterol; CI, confidence interval; CRP, C‐reactive protein; FAT, total fat; HbA1c, glycated haemoglobin; LDL, low‐density lipoprotein; SD, standard deviation; TRE, time‐restricted eating/16:8 fasting group; TRG, triglycerides.

^a^ Paired *t*‐test.

^b^ *t*‐test.

### 3.5 Patient Reported Outcome Measurements and Adverse Events

Two participants in the TRE reported increased feelings of hunger. Between T1 and T2, two participants in the CG reported oral discomfort, two controls were more sensitive during measurements compared with baseline, and one control had an aphthous ulcer at T3. OHIP values decreased significantly by 3.26 (6.33) in BF only (intra‐*p*‐value_BF_ T1–T3 = 0.024) (Table 3).

## 4 Discussion

This single‐blinded, three‐arm clinical trial investigated the effects of two intermittent fasting protocols compared with a non‐fasting regular dietary regimen using the model of an experimentally induced gingivitis. Evidence of an anti‐inflammatory potential was observed in the focused outcome parameter, with a lesser increase in BOP in the Bahá'í group compared with the other groups. Additionally, GCF levels did not increase in either fasting group, accompanied by metabolic improvements observed primarily in the fasting groups.

The most pronounced periodontal and metabolic changes were observed in the BF. Bahá'í individuals exhibited longer fasting hours (day and night combined) and a more severe calorie restriction compared with the other groups. This may have promoted significant reductions in metabolic parameters such as body weight and blood pressure after just 9 days of fasting, as demonstrated in other studies (Koppold‐Liebscher et al. 2021). Interestingly, previous IF studies failed to demonstrate reductions in circulating inflammatory parameters (Varady et al. 2022). However, the present Bahá'í cohort exhibited a significant reduction in CRP after 9 days.

BOP in the BF increased by only one third compared with the other two groups, also indicating a potential anti‐inflammatory effect. Similar results have been demonstrated in animal models with rhesus monkeys under long‐term calorie restriction with a 30% kcal reduction without malnutrition: A 3‐month ligature‐induced periodontitis model led to significantly less PPD, CAL, BOP and plaque compared with the *ad libitum* controls (Branch‐Mays et al. 2008). Despite the prolonged and invasive nature of the intervention, with animals undergoing calorie restriction for 13–17 years, the present study demonstrated an immediate effect of IF after just 9 days, without a prior fasting period. Reynolds et al. (2009) documented significantly lower PDD in the long‐term CR rhesus male group compared with male controls, as well as decreased levels of IL‐8 and IL‐1β in GCF. However, this unambiguous effect could not be demonstrated in females. Also, Ebersole et al. (2008) observed gender‐specific effects of calorie restriction in monkeys.

Gender‐specific differences in fasting are known from human studies, as women have different fat distribution (Ross et al. 1994), immunological response (Giefing‐Kröll et al. 2015) and health understandings (Barnes et al. 2008), that may affect their participation rates in clinical trials, and have been observed in previous studies (Pappe et al. 2021). However, a separate analysis by gender could not find a gender‐specific effect on periodontal parameters (Table S2).

The present study is in line with our previous observational trial on prolonged fasting that led to a significant reduction in BOP and GCF values after a 4‐ to 10‐day total fast in women with metabolic syndrome and periodontitis (Pappe et al. 2021). However, Squire and Costley (1976) reported a pro‐inflammatory effect in obese men undergoing a rigorous weight control program involving water‐only fasting for an average of 44 days (range: 15–71 days), supplemented with vitamins and minerals. This regimen resulted in decreased plaque and increased gingival index, potentially due to malnutrition and protein deficiency, and exceeding fasting time not aligning with recent fasting guidelines (De Wilhelmi Toledo et al. 2013). Similar findings were observed in studies on bariatric surgery after 6 months (Fontanille et al. 2018).

GCF values increased simultaneously with BOP in the CG, but not in the fasting groups. Park et al. (2015) also reported similar findings for GCF and BOP in a study involving 41 obese individuals who underwent a 4‐week high‐intensity physical training combined with calorie restriction. Although plaque levels increased significantly, the gingival index and BOP did not change. Additionally, pro‐inflammatory markers in GCF were significantly reduced compared with controls.

On the contrary, in a recent observational trial of 20 dental students with gingivitis practicing Ramadan, measurements of gingival index and plaque increased after 10 days of fasting (Narayanan et al. 2016). However, experimental gingivitis studies investigating the effect of anti‐inflammatory diets could document a reduction in BOP values after 4 weeks compared with controls (Bartha et al. 2022; Woelber et al. 2016, 2019). In the present cohorts, the dietary composition remained consistent throughout the study, potentially influencing periodontal inflammation due to its western diet characteristics, as revealed by the dietary questionnaires (Table S4). It is noteworthy that total fasting also exhibited a reducing effect, even or because of excluding all types of (unhealthy) foods. A recent study by Lira‐Junior et al. (2024) on IF in periodontal patients over 6 months supports these findings since IF was accompanied by a dietary change. This suggests that the effects of IF may be influenced by the consumed diet.

BOP_s values decreased upon reintroduction of oral hygiene but remained higher than T1 values in all groups, which were measured one week after PMPR. This demonstrates a failed short‐term effect of PMPR and underlines the strong habitual nature of cleaning practices (Weik et al. 2023).

## 5 Strengths and Limitations of the Study

The results for the BF are subject to greater scrutiny due to the absence of randomisation and the religious motivation (Ring et al. 2022). Also blinding of this group could not be fully ensured. The observed metabolic differences at T1 for the BF may have significantly impacted the outcomes. Self‐reported fasting revealed a mean of 14 h of fasting in the TRE, which could have compromised the outcome and questions the practicability of the intervention. Further, clinical measurements should start after an adjustment period of 2 weeks of IF protocols (Varady et al. 2022). The modified experimental approach of the ‘classic experimental gingivitis’ (Loe et al. 1965) was employed to enhance participants' participation rate. Options to improve data quality like using protective splints, extending the study period or omitting approximal hygiene could be considered in future studies (Dommisch et al. 2019; Woelber et al. 2019). Changing the test sextant did not affect plaque levels and, therefore, did not influence the outcome. The potential bias of two non‐calibrated examiners could be addressed through an additional ANCOVA and subsequent sub‐cohort analysis. Further, parameters such as diet, physical activity, alcohol consumption, ketone bodies, pro‐inflammatory markers in GCF, as well as the microbiota, and gender‐specific difference in fasting and induced gingivitis (Becerik et al. 2010) could be of interest for future studies.

## 6 Conclusion

During a modified experimental gingivitis model, BF resulted in lower BOP levels compared with TRE and CG, and GCF levels increased in the CG only. The effect may depend on fasting time and caloric intake, leading to greater metabolic reductions, such as body weight, CRP, blood pressure and HbA1c. The correlation between simultaneous increases in plaque and BOP needs to be clarified during different IF protocols. Additionally, GCF and BOP also do not appear to be reliable correlates during IF. Further investigation is needed to determine whether IF could be of therapeutic use for periodontal diseases.

## Author Contributions

C.L.P. was responsible for the study coordination, study design, recruitment, funding, data interpretation, and for writing the manuscript and the final version. J.M. and S.D. contributed to recruitment, data collection and critical review of the paper. F.K. was responsible for data analysis, interpretation and contributed to the critical reading of the manuscript. O.P.‐R., B.P., N.S. and A.M. were responsible for the critical reading of the manuscript. H.D. contributed to the study conduction, and was involved in data interpretation and critical review of the paper. D.K. provided the scientific fasting background and was involved in the study design and critical reading of the manuscript. All authors granted their approval for the final version of the paper and reached a consensus on all aspects of the work.

## Conflicts of Interest

D.K. and A.M. are members of the steering committee of the German Medical Association for Fasting and Nutrition (ÄGHE). D.K. has co‐founded the Academy of Integrative Fasting (AIF), an institution for the qualification of medical staff in clinical fasting applications. D.K. serves as a consultant for a mobile application on intermittent fasting (FASTIC) as well as a company producing plant‐based supplements (EVERYYIN). A.M. is also co‐founder of the SALUFAST company and serves as a consultant for Lanserhof.

## Supporting information


**Appendix S1.** Supporting information.

**Appendix S2.** Supporting information.

## Data Availability

The data that support the findings of this study are available from the corresponding author upon reasonable request.
