# Supplementary material for: Intermittent Fasting Regimes Reduce Gingival Inflammation: A Three‐Arm Clinical Trial
Source: J Clin Periodontol. 2025 Mar 9;52(5):681–94. doi: 10.1111/jcpe.14151 (PMC12003054; doi:10.1111/jcpe.14151)
Supplement: Supplementary file 1 — Appendix S1. Supporting information. [file JCPE-52-681-s001.docx]

**Appendix S1**

**Pre-treatment and experimental gingivitis model**

The screening and pre-treatment were scheduled one week prior the study begin (T0) and included the assessment of medical history, dental status/DMFT index (Klein, Palmer, & Knutson, 1938), periodontal screening index (Meyle & Jepsen, 2000) as well as a full mouth bleeding on probing (BOP) (Ainamo & Bay, 1975) and plaque control record (PCR) (O'Leary, Drake, & Naylor, 1972). If necessary, a subsequent professional mechanical plaque removal (PMPR) including oral hygiene instructions was performed to reduce gingival inflammation prior study begin using airscaler and scaler (KaVo Dental GmbH, Germany, Hu-Friedy Mfg. Co., LLC, German). To finish the entire dentition was polished (Proxyt® Rosa – RDA 7, Ivoclar Vivadent GmbH, Germany) and fluoridated (elmex® gelée, CP GABA GmbH, Germany).

The experimental gingivitis phase started after baseline measurements (T1) and lasted until the following examination after 9-10 days (T2). The duration was dependent on the participant’s personal schedule. (Figure 1). Subjects were instructed on where not to brush and perform interdental cleaning (test sextant teeth 24-27). If crowns or prosthetics were present in that area the opposite upper side was determined (teeth 14-17). A large red adhesive sticker was provided as a reminder on bathroom mirrors. Additionally, all participants received the same toothpaste (elmex® Kariesschutz professionel Zahnpasta, CP GABA GmbH, Germany) and were advised not to use additional mouth rinse, chewing gum, or to chew hard-textured foods on the test side during T1-T2. All female participants received a urine pregnancy test (hCG Ultra Test, MEXACARE GmbH, Germany) which was to be conducted directly before T1 to exclude pregnancy during the study.

**Fasting interventions**

Bahá’í fasting is a religiously motivated intermittent daytime dry fasting practice. Bahá’í abstain from consuming drink and food from sunrise until sunset. It is performed annually in March for 19 days, coinciding with the month when day and night durations are approximately equal (equinox) leading to a daytime fast of around 12 h. Food intake is typically reduced to early in the morning and evenings (Koppold-Liebscher et al., 2021). Subjects in the TRE group were permitted to consume isocaloric solid foods and beverages within an eight-hour window during the day, which they were free to choose. Outside of this window, only non-caloric beverages such as water or tea were allowed (Schuppelius, Peters, Ottawa, & Pivovarova-Ramich, 2021). Fasting interventions started after T1 measurements were taken and ended after 19 days (T3). The CG was told to continue with their habitual eating window. All participants were instructed not to alter the types of foods they typically consume during the study period and were asked to document their eating and fasting hours (T1-T3) (Figure 1).

**Oral and clinical examination**

The following parameters were assessed by two blinded experienced examiners (SL, SB) in all groups at all time points (T1-T3): full mouth BOP using a pressure calibrated probe (UNC15, Aesculap AG, Germany) and PCR using a fluorescent staining solution (Plaque Test indicator liquid, Ivoclar Vivadent GmbH, Germany. Additionally, rustogi plaque control record (RPI) was measured (Rustogi et al., 1992) and a BOP_s was separately calculated, both for the test sextant.

Gingival crevicular fluid (GCF) was assessed using the Periotron^©^ 8000 device (Berlin) and Periotron^©^ 6000 (Halle) (Oraflow Inc, USA). Four samples were collected for 30 seconds from both interproximal areas of the upper tested premolars under dry and cleaned conditions using PerioPaper® (Oralflow Inc). Under the use of cotton rolls, suction and teeth were air-dried ≥30 sec prior to sampling, if contaminated with blood or saliva sampling was repeated. The device was cleaned between measurements using 70% ethanol, dried, and calibrated with a dry test strip prior to each use. To prevent manipulation of GCF, participants refrained from chewing or drinking for 2 hours prior to the study. All appointments were scheduled in the morning (8-11 am) to minimize circadian bias (Griffiths, 2003). Mean values were calculated from the repeated measurements. Calibration data for both devices were conducted using NaCl solution with defined volumes (0.1-2.0 μl) and were exponentially interpolated to estimate calibrated sulcus fluid data from the raw measurements (GCF_ad). After GCF sampling, stimulated saliva was collected for 3 min using a paraffin block (Ortho Wachs, Wellsamed GmbH, Germany) and pH level were determined using a calibrated scale (MK-2000B Petite Balance, YMC, Japan) and pH test strips (pH-Fix 0-14, Carl Roth GmbH + Co. KG; Germany), respectively.

Metabolic parameters were assessed at T1-T3. Blood pressure was measured in sitting position (BM40, Beurer GmbH, Germany). Waist circumference (WC) and hip circumference (HC) were quantified with the upper body unrestricted, while standing, using a flexible tape measure. Body weight (BW), BMI, and body composition (fat distribution and muscle mass) were assessed using a bioimpedance analyzer (BF508, Omron Medizintechnik, Germany). Measurements were conducted after emptying the bladder and without heavy clothing. Age, height, and sex were recorded prior to measurements. After disinfection (Octenisept®, Schülke & Mayr GmbH, Germany) 20 ml blood samples were collected (BD Vacutainer®, BD, USA) and analyzed (Labor Berlin – Charité Vivantes GmbH, Germany) for c-reactive protein (CRP), Interleukin-6 (IL-6), total cholesterol (CHOL), triglycerides (TRG), high lipoprotein (HDL), low-density lipoprotein (LDL) and glycated hemoglobin (HbA1c) (Figure 1).

The change in BOP_s between T1 and T2 was considered as primary outcome, while other parameter changes were secondary outcomes.

At each time point (T1-T3), oral health-related quality of life was assessed using the Oral Health Impact Profile (OHIP-21) (Figure 1). Responses were collected using a web-based survey tool hosted by the Department of Internal Medicine and Nature-Based Therapies, Charité (nhk.charite.de/limesurvey; Version 5.6, 2022). A subgroup of participants received continuous glucose sensors after T0 and were required to document their food intake throughout the study period (Peters et al., 2024).
