# Supplementary material for: Intermittent Fasting Regimes Reduce Gingival Inflammation: A Three‐Arm Clinical Trial
Source: J Clin Periodontol. 2025 Mar 9;52(5):681–94. doi: 10.1111/jcpe.14151 (PMC12003054; doi:10.1111/jcpe.14151)
Supplement: Supplementary file 2 — Appendix S2. Supporting information. [file JCPE-52-681-s002.docx]

Appendix S2

Table S1 Total and overnight fasting time is given for both fasting groups and controls (T1 -T2 and T1 -3 in parentheses).

|  |  |  |  |  |  |  |  | **TRE-CG** | **BF-CG** | **BF-TRE** |
| --- | --- | --- | --- | --- | --- | --- | --- | --- | --- | --- |
| **Fasting** | | **T1** | **T2** |  | **T1-T2** | |  | **𝝙 T2-T1** | | |
| **Time** | |  |  |  |  | intra- |  | [CI] | | |
|  |  | mean (SD) | mean (SD) |  | Diff. (SD) | p value* |  | inter-p value** | | |
| fasting night | TRE | 11.32 (2.34) | 14.86 (3.4) |  | 3.55 (4.27) | 0.001 |  | 3.97 | -0.35 | -4.33 |
| (h/day) | BF | 11.04 (2.77) | 10.26 (3.3) |  | -0.78 (4.94) | 0.466 |  | [1.94; 6.00] | [-2.62; 1.91] | [-7.17; -1.49] |
|  | CG | 11.71 (1.52) | 11.29 (1.39) |  | -0.43 (1.47) | 0.206 |  | **<0.001** | 0.751 | **0.004** |
| fasting total | TRE | 11.32 (2.34) | 14.86 (3.4) |  | 3.55 (4.27) | 0.001 |  | 3.970 | 5.55 | 1.57 |
| (h/day) | BF | 11.04 (2.77) | 16.16 (4.58) |  | 5.12 (5.37) | 0.0001 |  | [1.94; 6.00] | [3.10; 8.00] | [-1.41; 4.55] |
|  | CG | 11.71 (1.52) | 11.29 (1.39) |  | -0.43 (1.47) | 0.206 |  | **<0.001** | **0.0001** | 0.293 |
|  |  | **T1** | **T3** |  | **T1-T3** | |  | **𝝙 T1-T3** | | |
| fasting night | TRE | 11.32 (2.34) | 14.55 (3.34) |  | 3.23 (3.84) | <0.001 |  | 3.32 | -1.25 | -4.58 |
| (h/day) | BF | 11.04 (2.77) | 9.7 (1.88) |  | -1.35 (3.32) | 0.069 |  | [1.49; 5.16] | [-2.83; 0.33] | [-6.79; -2.36] |
|  | CG | 11.71 (1.52) | 11.62 (1.25) |  | -0.1 (1.41) | 0.766 |  | **<0.001** | 0.116 | 0.0001 |
| fasting total | TRE | 11.32 (2.34) | 14.55 (3.34) |  | 3.23 (3.84) | <0.001 |  | 3.32 | 5.53 | 2.21 |
| (h/day) | BF | 11.04 (2.77) | 16.48 (4.32) |  | 5.43 (4.64) | <0.0001 |  | [1.49; 5.16] | [3.40; 7.66] | [-0.41; 4.82] |
|  | CG | 11.71 (1.52) | 11.62 (1.25) |  | -0.1 (1.41) | 0.766 |  | **<0.001** | **<0.0001** | 0.096 |

Abbreviations: BF, Bahá’í fasting group; CG, control group; CI, confidence interval; SD, standard deviation; TRE, time-restricted eating/ 16:8 fasting group.

* paired t-test

** t-test

Table S2 Analysis by gender and center for primary outcome bleeding on probing and gingival crevicular fluid (only T1 and T2 in parentheses).

|  |  |  |  |  |  |  |  |  |  |  | **TRE-CG** | **BF-CG** | **BF-TRE** |
| --- | --- | --- | --- | --- | --- | --- | --- | --- | --- | --- | --- | --- | --- |
| **Analysis by** | | | | **T1** | **T2** |  | **T1-T2** | | |  | **𝝙 T2-T1** | | |
| **gender and clinic** | | | |  |  |  |  |  | intra- |  | [CI] | | |
|  |  | n |  | mean (SD) | mean (SD) |  | Diff. | [CI] | p value* |  | inter-p value** | | |
| BOP_s (%) | TRE | 16 | f | 7.25 (6.49) | 21.08 (13.62) |  | 13,83 | [7.56; 20.10] | **0.0006** |  | -4,65 | -11,68 | -7,03 |
|  | BFG | 11 | f | 5.31 (4) | 12.11 (9.13) |  | 6,8 | [1.61; 11.99] | **0.028** |  | [-16.25; 6.96] | [-22.78; -0.58] | [-15.58; 1.52] |
|  | CG | 14 | f | 7.69 (4.93) | 26.17 (18.61) |  | 18,48 | [9.42; 27.53] | **0.0015** |  | 0.416 | **0.04** | 0.102 |
|  | TRE | 6 | m | 11.72 (7.02) | 28.41 (8.09) |  | 16,69 | [11.19; 22.19] | **0.002** |  | 8,95 | -3,59 | -12,54 |
|  | BFG | 12 | m | 4.17 (4.81) | 8.32 (5.36) |  | 4,15 | [0.53; 7.76] | **0.046** |  | [-0.63; 18.54] | [-12.10; 4.92] | [-20.08; -5.00] |
|  | CG | 7 | m | 5.93 (5.37) | 13.67 (10.37) |  | 7,74 | [1.21; 14.26] | **0.059** |  | 0.064 | 0.368 | **0.004** |
| BOP_s (%) | TRE | 14 | B | 9.76 (7.49) | 29.16 (10.08) |  | 19,4 | [14.99; 23.81] | **<0.001** |  | -0,87 | -10,45 | -9,59 |
|  | BFG | 11 | B | 6.83 (4.07) | 16.64 (5.34) |  | 9,81 | [5.37; 14.25] | **0.0015** |  | [-11.04; 9.31] | [-20.83; -0.08] | [-16.20; -2.97] |
|  | CG | 14 | B | 8.89 (4.66) | 29.15 (16.82) |  | 20,26 | [11.62; 28.91] | **0.0005** |  | 0.862 | **0.048** | **0.006** |
|  | TRE | 8 | H | 6.21 (5.09) | 12.45 (9.68) |  | 6,23 | [-1.73; 14.19] | 0.169 |  | 2,07 | -2,78 | -4,85 |
|  | BFG | 12 | H | 2.77 (3.92) | 4.16 (3.4) |  | 1,39 | [-1.51; 4.29] | 0.368 |  | [-7.90; 12.04] | [-7.83; 2.28] | [-14.64; 4.95] |
|  | CG | 7 | H | 3.54 (4.12) | 7.71 (5.64) |  | 4,17 | [0.59; 7.74] | 0.062 |  | 0.652 | 0.257 | 0.682 |
| GCF_ad (ul) | TRE | 16 | f | 0.18 (0.13) | 0.16 (0.15) |  | -0,02 | [-0.09; 0.05] | 0.618 |  | -0,13 | -0,09 | 0,04 |
|  | BFG | 11 | f | 0.26 (0.1) | 0.28 (0.1) |  | 0,02 | [-0.10; 0.14] | 0.707 |  | [-0.24; -0.03] | [-0.24; 0.06] | [-0.11; 0.19] |
|  | CG | 14 | f | 0.11 (0.11) | 0.23 (0.15) |  | 0,11 | [0.04; 0.18] | **0.008** |  | **0.016** | 0.219 | 0.561 |
|  | TRE | 6 | m | 0.09 (0.06) | 0.16 (0.12) |  | 0,07 | [-0.02; 0.15] | 0.179 |  | 0,03 | -0,01 | -0,04 |
|  | BFG | 12 | m | 0.24 (0.17) | 0.27 (0.13) |  | 0,03 | [-0.09; 0.15] | 0.646 |  | [-0.10; 0.16] | [-0.16; 0.15] | [-0.20; 0.12] |
|  | CG | 7 | m | 0.2 (0.15) | 0.23 (0.07) |  | 0,04 | [-0.05; 0.12] | 0.426 |  | 0.623 | 0.932 | 0.633 |

Abbreviations: B, Berlin; BF, Bahá’í fasting group; BOP_s, bleeding n probing in test sextant (24-27); CG, control group; CI, confidence interval; f, female; GCF, gingival crevicular fluid; H, Halle; m, male; SD, standard deviation; TRE, time-restricted eating/ 16:8 fasting group.

* paired t-test

** t-test

Table S3 Periodontal parameters for both fasting groups and controls (only T0 and T3 in parentheses).

|  |  |  |  |  |  |  |
| --- | --- | --- | --- | --- | --- | --- |
| **Periodontal** | | **T0** | **T3** |  | **T3-T0** | |
| **parameters** | |  |  |  |  | intra- |
|  |  | mean (SD) | mean (SD) |  | Diff. (SD) | p value* |
| BOP_s | TRE | 9.27 (7.79) | 15.05 (9.82) |  | 5.78 (11.84) | 0.04 |
|  | BF | 9.22 (8.42) | 10.72 (7.48) |  | 1.07 (8.99) | 0.57 |
|  | CG | 12.86 (9.86) | 13.86 (11.67) |  | 0.99 (11.32) | 0.69 |
| BOP | TRE | 7.89 (5.63) | 11.51 (9.37) |  | 3.62 (9.79) | 0.11 |
|  | BF | 10.34 (10.92) | 6.73 (4.27) |  | -3,61 (8.94) | 0.06 |
|  | CG | 10.31 (7.94) | 8.83 (5.85) |  | -1.48 (5.03) | 0.19 |
| PCR | TRE | 31.58 (17.51) | 25.97 (17.74) |  | -5.61 (16.42) | 0.13 |
|  | BF | 42.44 (18.3) | 31.29 (12.86) |  | -11.15 (20.83) | 0.02 |
|  | CG | 32.11 (15.22) | 24.56 (16.16) |  | -7.56 (20.9) | 0.11 |

Abbreviations: BF, Bahá’í fasting group; BOP, bleeding on probing; BOP_s, bleeding n probing in test sextant (24-27), CG, control group; SD, standard deviation; PCR, plaque control record.

* paired t-test

Table S4 Consumed food at Baseline based on self-reported food frequency questionnaires

| **consumed** |  | **Bahá'í Fasting** | **16:8 Fasting** | **Control** |
| --- | --- | --- | --- | --- |
| **food per day at T1** |  | **Group (BF)** | **Group (TRE)** | **Group (CG)** |
|  |  | MW (SD) | MW(SD) | MW (SD) |
|  |  |  |  |  |
| Vegetables | 1 portion (100 g) | 1.42 (0.75) 1.76 (0.79) 1.61 (0.78) | | |
| Fruits | 1 portion (100 g) | 1.16 (0.84) 1.23 (0.78) 1.01 (0.76) | | |
| Cold Cuts | 1 portion (25 g) | 0.23 (0.45) 0.17 (0.27) 0.24 (0.47) | | |
| Meat | 1 portion (175 g) | 0.38 (0.44) 0.22 (0.26) 0.20 (0.28) | | |
| Cheese | 1 portion (25 g) | 0.71 (0.45) 0.78 (0.57) 0.86 (0.79) | | |
| Sweets | times/day | 0.83 (0.47) 0.85 (0.54) 1.11 (0.76) | | |
| Fastfood | times/day | 0.24 (0.23) 0.17 (0.16) 0.19 (0.17) | | |
| Softdrinks | 1 glass (0,33 L) | 0.54 (0.34) 0.25 (0.29) 0.24 (0.27) | | |

| **Question** | **Answer and Choices** |
| --- | --- |
| What best describes your usual diet? | With meat and fish |
|  | With meat without fish |
|  | Without meat but with fish |
|  | Vegetarian with dairy products and eggs |
|  | Vegetarian with dairy products, without eggs |
|  | Vegan without animal products |
|  | Other: |
| How many meals (including snacks) did you consume on average per day in the last week? | One meal |
|  | Two meals |
|  | Three meals |
|  | Four meals |
|  | Five meals |
|  | More than five meals |
| Did you tend to snack between main meals in the last week? (e.g., chocolate bars, sweets, pastries, chips, etc.) | Yes |
|  | No |
| How many hours on average passed between your last meal and your first meal the next day in the last week? | *[Numerical entry]* |
| How many portions of vegetables did you eat approximately last week? | 3 portions daily or more |
|  | 2 portions daily |
|  | 1 portion daily |
|  | 5-6 portions per week |
|  | 3-4 portions per week |
|  | 1-2 portions per week |
|  | Rarely or not at all |
| How many portions of fruit did you eat approximately last week? | 3 portions daily or more |
|  | 2 portions daily |
|  | 1 portion daily |
|  | 5-6 portions per week |
|  | 3-4 portions per week |
|  | 1-2 portions per week |
|  | Rarely or not at all |
| How many portions of sausage did you eat approximately last week? | 3 portions daily or more |
|  | 2 portions daily |
|  | 1 portion daily |
|  | 5-6 portions per week |
|  | 3-4 portions per week |
|  | 1-2 portions per week |
|  | Rarely or not at all |
| How many portions of meat did you eat approximately last week? | 3 times daily or more |
|  | 2 times daily |
|  | 1 time daily |
|  | 5-6 times per week |
|  | 3-4 times per week |
|  | 1-2 times per week |
|  | Rarely or not at all |
| How many portions of cheese did you eat approximately last week? | 3 portions daily or more |
|  | 2 portions daily |
|  | 1 portion daily |
|  | 5-6 portions per week |
|  | 3-4 portions per week |
|  | 1-2 portions per week |
|  | Rarely or not at all |
| How often did you eat sweets last week? (e.g., cakes, ice cream, chocolate, etc.) | 3 times daily or more |
|  | 2 times daily |
|  | 1 time daily |
|  | 5-6 times per week |
|  | 3-4 times per week |
|  | 1-2 times per week |
|  | Rarely or not at all |
| How often did you eat fast food last week? (e.g., fries, burgers, pizza, bratwurst) | 3 times daily or more |
|  | 2 times daily |
|  | 1 time daily |
|  | 5-6 times per week |
|  | 3-4 times per week |
|  | 1-2 times per week |
|  | Rarely or not at all |
| How many glasses of sweetened soft drinks or juices did you drink last week? | 3 glasses daily or more |
|  | 2 glasses daily |
|  | 1 glass daily |
|  | 5-6 glasses per week |
|  | 3-4 glasses per week |
|  | 1-2 glasses per week |
|  | Less or none |
| How often did you use whole grain products last week? (e.g., bread, rice, pasta) | Exclusively |
|  | Mostly |
|  | About half the time |
|  | Occasionally |
|  | Rarely or not at all |

Table S5 ANOVA calculation for all parameters (main factors group and visit and their interaction)

| **Parameter** | **Source** | **DF1** | **DF2** | **F** | **p** | **np2** | **p-corr** |
| --- | --- | --- | --- | --- | --- | --- | --- |
| BW | group | 2 | 61 | 1.408 | 0.253 | 0.044 |  |
|  | visit | 2 | 122 | 4.556 | 0.012 | 0.069 | 0.036 |
|  | Interaction | 4 | 122 | 2.036 | 0.094 | 0.063 |  |
|  |  |  |  |  |  |  |  |
| BMI | group | 2 | 63 | 0.605 | 0.549 | 0.019 |  |
|  | visit | 2 | 126 | 6.871 | 0.001 | 0.098 | 0.011 |
|  | Interaction | 4 | 126 | 3.212 | 0.015 | 0.093 |  |
|  |  |  |  |  |  |  |  |
| BP_sys | group | 2 | 63 | 0.146 | 0.864 | 0.005 |  |
|  | visit | 2 | 126 | 3.577 | 0.031 | 0.054 |  |
|  | Interaction | 4 | 126 | 3.441 | 0.011 | 0.098 |  |
|  |  |  |  |  |  |  |  |
| BP_dia | group | 2 | 63 | 1.743 | 0.183 | 0.052 |  |
|  | visit | 2 | 126 | 0.282 | 0.755 | 0.004 |  |
|  | Interaction | 4 | 126 | 0.676 | 0.610 | 0.021 |  |
|  |  |  |  |  |  |  |  |
| FAT_vis | group | 2 | 63 | 1.146 | 0.325 | 0.035 |  |
|  | visit | 2 | 126 | 3.970 | 0.021 | 0.059 | 0.042 |
|  | Interaction | 4 | 126 | 2.156 | 0.078 | 0.064 |  |
|  |  |  |  |  |  |  |  |
| FAT_ges | group | 2 | 63 | 0.175 | 0.840 | 0.006 |  |
|  | visit | 2 | 126 | 3.213 | 0.044 | 0.049 | 0.064 |
|  | Interaction | 4 | 126 | 1.208 | 0.311 | 0.037 |  |
|  |  |  |  |  |  |  |  |
|  |  |  |  |  |  |  |  |
| **Parameter** | **Source** | **DF1** | **DF2** | **F** | **p** | **np2** | **p-corr** |
| GCF | group | 2 | 63 | 3.407 | 0.039 | 0.098 |  |
|  | visit | 2 | 126 | 15.727 | 0.000 | 0.200 |  |
|  | Interaction | 4 | 126 | 2.554 | 0.042 | 0.075 |  |
|  |  |  |  |  |  |  |  |
| PCR | group | 2 | 63 | 1.595 | 0.211 | 0.048 |  |
|  | visit | 2 | 126 | 57.189 | 0.000 | 0.476 | 0.000 |
|  | Interaction | 4 | 126 | 0.240 | 0.915 | 0.008 |  |
|  |  |  |  |  |  |  |  |
| BOP_s | group | 2 | 63 | 4.238 | 0.019 | 0.119 |  |
|  | visit | 2 | 126 | 45.376 | 0.000 | 0.419 | 0.000 |
|  | Interaction | 4 | 126 | 4.470 | 0.002 | 0.124 |  |
|  |  |  |  |  |  |  |  |
| Saliva | group | 2 | 63 | 0.312 | 0.733 | 0.010 |  |
|  | visit | 2 | 126 | 2.755 | 0.067 | 0.042 | 0.075 |
|  | Interaction | 4 | 126 | 1.259 | 0.290 | 0.038 |  |
|  |  |  |  |  |  |  |  |
| ph | group | 2 | 63 | 0.348 | 0.708 | 0.011 |  |
|  | visit | 2 | 126 | 0.752 | 0.474 | 0.012 |  |
|  | Interaction | 4 | 126 | 1.093 | 0.363 | 0.034 |  |
|  |  |  |  |  |  |  |  |
| RPI | group | 2 | 63 | 0.596 | 0.554 | 0.019 |  |
|  | visit | 2 | 126 | 387.024 | 0.000 | 0.860 | 0.000 |
|  | Interaction | 4 | 126 | 0.083 | 0.988 | 0.003 |  |
|  |  |  |  |  |  |  |  |
| CHOL | group | 2 | 63 | 1.017 | 0.368 | 0.031 |  |
| l | visit | 2 | 126 | 5.195 | 0.007 | 0.076 | 0.012 |
|  | Interaction | 4 | 126 | 3.929 | 0.005 | 0.111 |  |
|  |  |  |  |  |  |  |  |
| TRG | group | 2 | 63 | 2.084 | 0.133 | 0.062 |  |
|  | visit | 2 | 126 | 1.710 | 0.185 | 0.026 |  |
|  | Interaction | 4 | 126 | 1.118 | 0.351 | 0.034 |  |
| **Parameter** | **Source** | **DF1** | **DF2** | **F** | **p** | **np2** | **p-corr** |
| HDL | group | 2 | 63 | 4.751 | 0.012 | 0.131 |  |
|  | visit | 2 | 126 | 2.213 | 0.114 | 0.034 |  |
|  | Interaction | 4 | 126 | 3.109 | 0.018 | 0.090 |  |
|  |  |  |  |  |  |  |  |
| LDL | group | 2 | 63 | 2.762 | 0.071 | 0.081 |  |
|  | visit | 2 | 126 | 4.729 | 0.010 | 0.070 |  |
|  | Interaction | 4 | 126 | 3.217 | 0.015 | 0.093 |  |
|  |  |  |  |  |  |  |  |
| CRP | group | 2 | 63 | 2.231 | 0.116 | 0.066 |  |
|  | visit | 2 | 126 | 4.173 | 0.018 | 0.062 | 0.023 |
|  | Interaction | 4 | 126 | 0.527 | 0.716 | 0.016 |  |
|  |  |  |  |  |  |  |  |
| HbA1c | group | 2 | 63 | 0.773 | 0.466 | 0.024 |  |
|  | visit | 2 | 126 | 4.252 | 0.016 | 0.063 | 0.019 |
|  | Interaction | 4 | 126 | 0.694 | 0.598 | 0.022 |  |
|  |  |  |  |  |  |  |  |
| OHIP | group | 2 | 63 | 0.070 | 0.933 | 0.002 |  |
|  | visit | 2 | 126 | 2.234 | 0.111 | 0.034 | 0.122 |
|  | Interaction | 4 | 126 | 1.676 | 0.160 | 0.051 |  |

Abbreviations: BOP_s, bleeding on probing in test sextant (24-27); BMI, body mass index; BW, body weight; BP_sys, systolic blood pressure; CRP, C-reactive protein; DF, degree of freedom; F, test statistics of the ANOVA; FAT_v, visceral fat; GCF, gingival crevicular fluid; HbA1c, glycated hemoglobin; HDL, high density lipoprotein; LDL, low density lipoprotein; np2, effect size eta-squared; OHIP, oral health impact profile; p, significant value; p-Corr, corrected p-value for cases only in which data were not normally distributed; PCR, plaque control record; RPI, rustogi plaque index; TRG, triglycerides.

Table S6 ANCOVA calculation for T2

| **Parameter** | **Time** | **Source** | **DF** | **F** | **p** | **np2** |
| --- | --- | --- | --- | --- | --- | --- |
| BW | T2 | group | 2 | 2.883 | 0.064 | 0.088 |
| BMI | T2 | group | 2 | 3.834 | 0.027 | 0.110 |
| BP_sys | T2 | group | 2 | 4.053 | 0.022 | 0.116 |
| BP_dia | T2 | group | 2 | 1.092 | 0.342 | 0.034 |
| FAT_v | T2 | group | 2 | 1.062 | 0.352 | 0.033 |
| FAT | T2 | group | 2 | 1.328 | 0.272 | 0.041 |
| GCF | T2 | group | 2 | 2.932 | 0.061 | 0.086 |
| PCR | T2 | group | 2 | 1.241 | 0.296 | 0.038 |
| BOP_s | T2 | group | 2 | 4.073 | 0.022 | 0.116 |
| Saliva | T2 | group | 2 | 1.132 | 0.329 | 0.035 |
| ph | T2 | group | 2 | 1.460 | 0.240 | 0.045 |
| RPI | T2 | group | 2 | 0.158 | 0.854 | 0.005 |
| CHOL | T2 | group | 2 | 1.960 | 0.149 | 0.059 |
| TRG | T2 | group | 2 | 0.373 | 0.690 | 0.012 |
| HDL | T2 | group | 2 | 5.303 | 0.007 | 0.146 |
| LDL | T2 | group | 2 | 3.161 | 0.049 | 0.093 |
| CRP | T2 | group | 2 | 0.747 | 0.478 | 0.024 |
| HbA1c | T2 | group | 2 | 0.186 | 0.831 | 0.006 |
| OHIP | T2 | group | 2 | 0.537 | 0.587 | 0.017 |

Abbreviations: BOP_s, bleeding on probing in test sextant (24-27); BMI, body mass index; BW, body weight; BP_sys/dia, systolic/diastolic blood pressure; CHOL, total cholesterol; CRP, C-reactive protein; DF, degree of freedom; F, test statistics of the ANCOVA; FAT, total fat; FAT_v, visceral fat; GCF, gingival crevicular fluid; HbA1c, glycated hemoglobin; HDL, high density lipoprotein; LDL, low density lipoprotein; np2, effect size eta-squared; OHIP, oral health impact profile; PCR, plaque control record; RPI, rustogi plaque index; TRG, triglycerides.
